# Lymphocyte Depletion in Experimental Hemorrhagic Shock in Swine

**DOI:** 10.1186/1476-9255-9-34

**Published:** 2012-09-25

**Authors:** Jason S Hawksworth, Christopher Graybill, Trevor S Brown, Suzanne M Gillern, Shannon M Wallace, Thomas A Davis, Eric A Elster, Doug K Tadaki

**Affiliations:** 1 Regenerative Medicine Department, Operational and Undersea Medicine Directorate, Naval Medical Research Center, Silver Spring, MD 20910, USA; 2 Department of Surgery, Walter Reed National Military Medical Center, Bethesda, MD, 20889, USA; 3 Norman M. Rich Department of Surgery, Uniformed Services University of Health Sciences, Bethesda, MD 20814, USA; 4 Department of Comparative Pathology, Walter Reed National Military Medical Center, Bethesda, MD, 20889, USA

**Keywords:** Lymphocyte depletion, Anti-thymocyte globulin, Combat casualty, Swine, Liver injury, Hemorrhagic shock

## Abstract

**Background:**

Hemorrhagic shock results in systemic activation of the immune system and leads to ischemia-reperfusion injury. Lymphocytes have been identified as critical mediators of the early innate immune response to ischemia-reperfusion injury, and immunomodulation of lymphocytes may prevent secondary immunologic injury in surgical and trauma patients.

**Methods:**

Yorkshire swine were anesthetized and underwent a grade III liver injury with uncontrolled hemorrhage to induce hemorrhagic shock. Experimental groups were treated with a lymphocyte depletional agent, porcine polyclonal anti-thymocyte globulin (PATG) (n = 8) and compared to a vehicle control group (n = 9). Animals were observed over a 3 day survival period. Circulating lymphocytes were examined with FACS analysis for CD3/CD4/CD8, and central lymphocytes with mesenteric lymph node and spleen staining for CD3. Circulating and lung tissue16 infiltrating neutrophils were measured. Circulating CD3 lymphocytes in the blood and in central lymphoid organs (spleen/lymph node) were stained and evaluated using FACS analysis. Immune-related gene expression from liver tissue was quantified using RT-PCR.

**Results:**

The overall survival was 22% (2/9) in the control and 75% (6/8) in the PATG groups, p = 0.09; during the reperfusion period (following hemorrhage) survival was 25% (2/8) in the control and 100% (6/6) in the PATG groups, p = 0.008. Mean blood loss and hemodynamic profiles were not significantly different between the experimental and control groups. Circulating CD3^+^CD4^+^ lymphocytes were significantly depleted in the PATG group compared to control. Lymphocyte depletion in the setting of hemorrhagic shock also significantly decreased circulating and lung tissue infiltrating neutrophils, and decreased expression of liver ischemia gene expression.

**Conclusions:**

Lymphocyte manipulation with a depletional (PATG) strategy improves reperfusion survival in experimental hemorrhagic shock using a porcine liver injury model. This proof of principle study paves the way for further development of immunomodulation approaches to ameliorate secondary immune injury following hemorrhagic shock.

## Background

The immune system is teleologically designed to respond only to local injury and is necessary for hemostasis, protection against microorganism invasion, and initiation of tissue repair. Severe trauma and hemorrhagic shock induce a systemic inflammatory response that results in inappropriate immune activation and secondary injury to the host [1]. Systemic inflammatory intensification following injury manifests clinically as the systemic inflammatory response syndrome, acute respiratory distress syndrome, and ultimately multi-organ dysfunction syndrome manifests clinically from SIRS, to ARDS, and ultimately to MOD [2]. Modulation of the immunologic response to injury is a major therapeutic goal to reduce associated morbidity and mortality and improve trauma outcomes.

The majority of research on clinical therapeutics for ischemia reperfusion injury has focused on monocyte and neutrophil adhesion blockade. However, despite promising preclinical data, results of phase 2 and 3 trials of neutrophil anti-adhesion therapy in ischemia-reperfusion injury (IRI) disorders have been disappointing [3]. In two clinical trials testing humanized CD18 monoclonal antibodies in the setting of traumatic injury, mortality and other primary end points were not significantly affected [4,5]. These failures are likely the result of the redundancy of adhesion pathways, but also suggest that the neutrophil is not central in the innate immune response to IRI.

Lymphocytes have recently been identified as critical mediators of the early innate immune response to injury. There is emerging evidence that T lymphocytes are rapidly activated in an alloantigen-independent manner in the setting of IRI [6,7]. Hypoxia is thought to be sufficient for lymphocyte activation, as CD4^+^ lymphocytes have been shown to increase adhesion to endothelial monolayers following anoxia modulation [8,9]. Furthermore, lymphocytes have been shown to rapidly accumulate in target organs following ischemia and may represent very early cellular mediators of reperfusion injury [7]. The role of T lymphocytes as critical cellular mediators of the innate immune response to IRI has been corroborated in multiple IRI models [10-14]. In a renal IRI model, genetically engineered mice deficient in both CD4^+^ and CD8^+^ lymphocytes had substantially less kidney dysfunction after renal ischemia than did wild-type control mice [15]. Mice deficient in CD4^+^ and CD8^+^ lymphocytes also demonstrated less tissue neutrophil infiltration, suggesting that lymphocytes orchestrate cell-mediated innate responses to ischemia. Altogether, these studies demonstrate a novel, innate function of lymphocytes in the setting of ischemic injury. Thereby, immunomodulation of lymphocytes may offer a novel approach to attenuate detrimental immune responses to severe traumatic injury.

Antithymocyte globulin (ATG) is a potent lymphocyte depleting agent that is used clinically in induction and antirejection therapy for solid organ transplantation, treatment of graft versus host disease, and selected autoimmune diseases [16,17]. The primary mechanism of immunosuppression involves massive peripheral and central lymphocyte depletion primarily by complement and Fas/Fas-L mediated apoptosis pathways [18-20]. In addition, ATG results in antibody inhibition of nondepleted T lymphocytes and functional alteration of several membrane receptors (TCR/CD3) and coreceptors (CD2, CD4, and CD8) [18,21]. Given the innate role of the lymphocyte in IRI, ATG may effectively modulate the post-traumatic inflammatory response.

In this study, we investigated a lymphocyte depletion strategy in a large animal liver injury shock model. We developed a porcine specific ATG in order to determine if lymphocyte depletion improves survival following experimental hemorrhagic shock.

## Methods

The experiments reported herein were conducted according to the principles set forth in the “Guide for the Care and Use of Laboratory Animals,” Institute of Laboratory Animals Resources, National Research Council, National Academy Press, 2011. The study was approved by the National Medical Research Center Institutional Animal Care and Use Committee (IACUC) and all procedures were performed in animal facilities approved by the Association for Assessment and Accreditation for Laboratory Animal Care International (AAALAC).

### Development of porcine anti-thymocyte globulin (PATG)

Porcine thymuses were obtained aseptically from normal unmanipulated control pigs, and single-cell suspensions were prepared by gentle pressing thorough a nylon filter mesh (Tetko, Inc, Elmsford, NY USA) into cold RPMI 1640 medium (GIBCO, Grand Island, NY USA). Thymocytes were isolated on density gradients using Ficoll-Paque (Pharmacia Biotech, Uppsala, Sweden) to remove reds cells and granulocytes, washed three times and then resuspended in Dulbecco phosphate buffered saline (D-PBS; GIBCO). All cell preparations had a purity of >95% lymphocytes (data not shown).

To produce polyclonal antibody against porcine thymocytes, 125 adult female New Zealand White rabbits (Covance, Denver, PA USA) were immunized subcutaneously with 5 × 10^6^ purified porcine thymocytes in complete Freund’s adjuvant (Sigma, St. Louis, MO, USA). Subsequent intravenous booster immunizations of 5 × 10^6^ thymocytes were given on days 14, 28 and 42. The rabbits were terminally bled, by cardiac puncture, 7 days after the final immunization. The collected blood was pooled and sera isolated by centrifugation. The Ig fraction was purified using Protein G Sepharose-4 Fast Flow columns (Pharmacia), sterile filtered (0.2 μm) and stored at 4°C before use. All PATG used in these studies was obtained from a single batch and was tested to be endotoxin-low (<0.05 IU/mg) by QCL-1000 Chromogenic LAL (Cambrex BioWhitaker Biosciences, Walkersville, MD USA).

The titer of antiserum was tested by FACS analysis to measure antibody binding-coating of PATG to the cell surface of purified porcine thymocytes and peripheral blood leukocytes (gated lymphocyte, monocyte and granulocyte cell populations). Cells (1 × 10^6^/100 μl) were incubated at 4°C with PATG at concentrations ranging from 0.01 μg/ml to 100 μg/ml, washed, and incubated with FITC-labeled goat anti-rabbit antibody (Jackson ImmunoResearch Laboratories, West Grove, PA USA). Results were compared to with cells incubated with the same concentrations of unspecific rabbit Ig. The PATG exhibited stronger titer to thymocytes and peripheral blood CD4^+^ T-cells (receptor sites saturated ≥ 0.5 μg/1 × 10^6^ cells) than to peripheral blood granulocytes and monocytes (100 to 300-fold greater; data not shown). We performed a dose–response experiment and determined that 4 PATG doses of 10 mg/kg 24 hours apart were necessary for >50% sustained lymphocyte depletion (data not shown).

### Animal preparation

Male and female 3–12 month Yorkshire (*Sus scrofa domestica*) swine weighing 25-35 kg were acquired from ABI Farms (Donsboro, PA). Feed was withheld 12 hours before surgery. Prior to surgery, animals were sedated and anesthesia induced with intramuscular ketamine hydrochloride (12–20 mg/kg) and xylazine (2.2 mg/kg) as well as atropine sulfate (0.05 mg/kg) to decrease tracheal secretions. Mask ventilation with Isoflurane (5.0%) was used to facilitate endotracheal intubation. Pigs were ventilated (Ohmeda 7800 series ventilator, Datex, Madison, WI) at 12–15 breaths/min and tidal volume 10 mL/kg. Anesthesia was maintained with Isoflurane (1.5-2.5%) in 21-25% O2.

Following adequate anesthesia the right external and internal jugular veins and carotid artery were isolated. A 9Fr dual-lumen, tunneled Hickman catheter was placed in the external jugular vein for drug infusion and blood collection during the survival period. A 9Fr introducer sheath was placed in the internal jugular vein and a 7.5Fr pulmonary artery catheter (PAC; Edwards Life Sciences, Irvine, CA) was inserted for continuous hemodynamic monitoring. An 18 G Angiocath was placed in the carotid artery and mean arterial pressure (MAP) was continuously transduced. A midline laparotomy was performed to expose the liver and isolate the left lateral lobe. Urine was collected via bladder catheterization. Rectal temperature was monitored continuously. Normothermia (37°C) was maintained with a warming device (Model 505, Bair Hugger, Augustine Medical, Eden Prairie, MN).

### Liver injury and resuscitation

**Figure 1:**
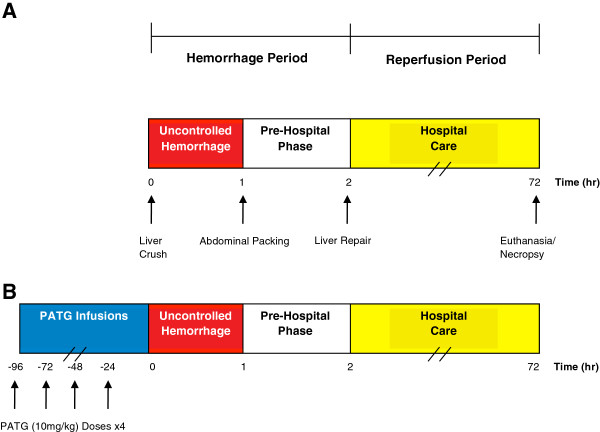
**Experimental design. A)** Liver injury was initiated at t = 0. Uncontrolled hemorrhage occurred until 1 hour, at which time the abdomen was packed and temporarily closed (pre-hospital phase). The animal was observed until 2 hours when hospital care was initiated. The liver was repaired and the abdomen definitively closed. The animal was then observed for a total of 72 hours. Blood transfusion was administered as indicated. Necropsy was performed when the animal expired or at 72 hours following euthanasia. The uncontrolled hemorrhage and pre-hospital phases were considered the hemorrhage period, while the hospital care phase was considered the reperfusion period. **B)** In the PATG experimental group, 4 doses of PATG (10 mg/kg) were administered starting 96 hours prior to liver injury.

All animal groups underwent a standardized liver injury and resuscitation protocol (Figure 1A). A reproducible liver injury was created by placing a ring clamp over the left lower lobe, 50% in width and 3.5-5 mm from the apex, adjusting for the relative size of the animal [22]. The clamp was closed and a 10 blade was used to lacerate the lobe from the top of the clamp through the remaining width. The liver injury denoted the start of the pre-hospital phase (Time 0). After 1 minute, the clamp was removed and the remaining tissue excised, resulting in a 25% lobectomy, consistent with a grade III liver injury. The hepatectomy specimen was weighed and divided by animal weight (kg) and reported as hepatectomy weight index (g/kg). Bleeding was spontaneous and removed continually via suction and quantified by weight. Blood loss weight (g) was used to calculate estimated blood loss volume (%). At 15 minutes, animals were infused 250 mL normal saline (NS) over the next 60 minutes. This time point represents the arrival of a first responder (e.g. medic) who has limited carrying capacity. After 1 hour blood collection was discontinued and the abdomen packed then closed with towel clips.

After a total of 2 hours, the pre-hospital phase was completed and hospital arrival was simulated. This time point represents arrival of the patient to a hospital with surgical capabilities. The abdomen was reopened, residual blood suctioned, sponges collected, and blood loss quantified by weight. The liver injury was repaired with suture and the abdomen definitively closed. Invasive lines were removed and the carotid artery repaired. One unit of allogeneic whole blood (10 mL/kg) (Thomas Morris, Reisterstown, MD) was administered for hemoglobin <7 g/dL, otherwise animals received additional NS at 10 mL/kg. Anesthesia was stopped and the animals extubated.

### Experimental group

Animals in the PATG group were anesthetized and underwent tunneled Hickman catheter placement 4 days prior to liver injury. PATG (10 mg/kg) was diluted in 250 mL NS and infused daily for 4 doses total (Figure 1B). Several animals experienced hemodynamic instability during the first infusion of PATG, likely secondary to cytokine release phenomena. Subsequently, the protocol was modified and one dose of Solumedrol (2 mg/kg) was administered prior to only the first PATG infusion which abrogated this drug effect.

### Post-surgical care

Pigs were allowed to eat a regular diet and drink water following surgery. Buprenorphine (0.05-0.1 mg/kg IM/IV) was administered every 6–12 hours if the animals demonstrated any sign of pain. If the pigs showed any sign of severe disability (e.g. inability to ambulate, eat, drink), severe infection, or uncontrolled pain, they were euthanized (Euthasol, Virbac AH, Fort Worth, TX) and taken for necropsy. At the end of the survival period (72 hours post-injury), surviving pigs were euthanized and taken for necropsy. Samples of the heart, lung, liver, kidney, small intestine, mesenteric lymph node, and spleen were taken. A portion of each sample was submitted for histology. Additional samples were placed in RNALater (Qiagen, Valencia, CA) and flash frozen using liquid nitrogen within 48 hours. These samples were stored at −80°C.

### Laboratory data

All functional laboratory assays were performed at 37°C, consistent with recorded normothermic animal temperatures (37.5 ±0.9°C). Blood samples were collected at 0, 15, 30, 60, 90, and 120 minutes during the hemorrhage period and at 18, 24, 36, 48, and 72 hours during the survival period. Complete blood count with differentiation was performed with a cell counter (NDvia120 Hematology System, Siemens, Deerfield, IL).

### Flow cytometry

Whole blood was fractionated using Histopaque 1077 (Sigma, St. Louis, MO) density gradient centrifugation. Fluorescent immunostaining of the isolated mononuclear cells was accomplished via a 30-minute incubation at 4°C with fluorescein isothiocyanate (FITC)-labeled CD3 and either phycoerythrin (PE)-labeled CD4 or CD8 mouse anti-porcine antibodies (BD Pharmingen, San Jose, CA). The cells were washed in FACS buffer (3% FBS, 1% sodium azide), fixed using 1.6% paraformaldehyde, and quantified by flow cytometry (Beckman-Coulter, Hialeah, FL). Appropriate mouse IgG isotype controls were used (BD Pharmingen, San Jose, CA). Flow cytometry analysis was performed with FACS Calibur (Becton Dickinson). Statistical analysis of the FACS data was performed with CellQuest (Becton Dickson).

### Immunohistochemistry

Tissue slides were deparaffinized using xylene followed by graded baths of ethanol. A DAKO Autostainer Plus Universal Staining System (DAKO, Carpenteria, CA) was used for automated immunohistochemical staining.

Immunohistochemical detection of CD3 was performed on sections of formalin fixed, paraffin embedded blocks of swine lymph node and spleen. Antigen retrieval was performed using Trilogy (Cell Marque, Rocklin, CA) for 30 minutes. Rat, anti- human CD3, monoclonal antibody (AbD Serotec, Raleigh, NC) was used at a dilution of 1:50 and incubated overnight at 4C. Biotinylated goat anti-rat IgG specific polyclonal antibody (BD Biosciences, San Jose, CA) was applied as a secondary antibody at a 1:100 dilution for 30 minutes at room temperature. The chromogen applied was 3, 3’ Diaminobenzidine (DAKO, Carpenteria, CA) for 10 minutes. The sections were counterstained with Hematoxylin (DAKO, Carpenteria, CA). External negative controls were processed identically as CD3 but the primary antibody was substituted with normal rat serum. A tissue sample was considered positive if reactive cells, lymphocytes, demonstrated reactivity. An Automated Cellular Imaging System (ACIS) was used to quantify the CD3 immunohistochemistry staining. By using the ACIS system, CD3 immunohistochemical staining could be specifically quantitated by determining how much brown (positive CD3 immunohistochemical reaction) was in the image compared to the amount of blue (hematoxylin nuclear counterstain).

Immunohistochemical detection of MPO was performed on sections of formalin fixed, paraffin embedded blocks of swine lung. Antigen retrieval was performed using Proteinase K (DAKO, Carpenteria CA) for 30 minutes. Polyclonal rabbit, anti- human MPO (DAKO, Carpenteria CA) was used at a ready to use dilution and incubated at room temperature for 30 minutes. Envision, anti-rabbit (DAKO, Carpenteria CA) was applied as a secondary antibody for 30 minutes at room temperature. The chromogen applied was 3, 3’ Diaminobenzidine (DAKO, Carpenteria, CA) for 10 minutes. The sections were counterstained with Hematoxylin (DAKO, Carpenteria, CA). External negative control was processed identically as with MPO but the primary antibody was substituted with normal rabbit serum. A tissue sample was considered positive if reactive cells, neutrophils, demonstrated reactivity in conjunction with a segmented nucleus. Neutrophils were noted mainly in circulation in small septal vessels and in large vessels. Cells with appropriate nuclear morphology and the presence of light to intensely reactive granules were counted; 5 random 40x fields were assessed.

### RNA extraction and quantitative real-time polymerase chain reaction

Total RNA was isolated from liver tissue taken at necropsy using Qiagen RNeasy Mini Kit (Qiagen Inc. Valencia, CA) according to manufacturer’s instructions. RNA purity and quantity were assessed by measuring the A_260_, A_280_, and A_230_on a Nanodrop Spectrophotometer (NanoDrop Technologies Inc. Wilmington, DE). RNA quality was determined from the 28S/18S rRNA ratio and RNA Integrity Number (RIN) using an Agilent 2100 BioAnalyzer (Agilent Technologies Inc. Santa Clara, CA). RIN values for all specimens in this study were ≥6.5. Reverse transcriptions were performed using Roche 1^st^ Strand Synthesis kits (Roche Diagnostics Corporation, Indianapolis, IN) according to the manufacturer’s protocol. Quantitative real-time polymerase chain reaction (QRT-PCR) was performed using the 7900HT Fast Real-Time PCR System (Applied Biosystems, Foster City, CA) to assess mRNA transcript expression of 21 immune-related genes. Taqman chemistry was used. Primers for 18S rRNA target were used as an internal control for each reaction. Primers and probes for the targets of interest were obtained from Applied Biosystems. All samples were run in duplicate. Individual samples were compared to averaged control tissue expression. Transcript quantification was derived using the comparative threshold cycle method [23] and reported as the median *n-*fold difference of the experimental sample to the control pool. Satisfactory mRNA was available for PATG (n = 6) was compared to the control (n = 5) group.

### Statistical analysis

Animals were randomly assigned to the control or experimental groups. Survival analysis between groups was performed by Kaplan Meier survival plots with the log rank test. Continuous data was compared with the Student *t-*test or nonparametric tests as appropriate. Multiple comparisons were performed with one-way ANOVA. A one-way ANOVA with repeated measures design was used for continuous time-dependent comparisons. Statistical analysis was performed using SPSS (SPSS Inc., Chicago, IL). A two-tailed p value < 0.05 was considered statistically significant. All data is represented as means ± standard error (SEM) unless otherwise specified.

## Results

### Animal characteristics and hemodynamic profiles during hemorrhage period

Mean animal weight and gender distribution were not significantly different between the control (n = 9) and PATG (n = 8) groups (Table 1). The mean hepatectomy weight index was similar between groups indicating that the liver injury was well standardized. In addition, the majority of animals in both groups required blood transfusion during the hospital phase for hemoglobin value <7 g/dL.

**Table 1:** Animal characteristics

**Variable**	**Control (n=9)**	**PATG (n=8)**	**p-value**
Mean animal weight [kg]	32.5 ±1.7	28.51 ±0.8	NS^1^
Animal gender - M:F	5:4	4:4	NS^2^
Mean hepatectomy weight index [g/kg]	0.31 ±0.04	0.33 ±0.03	NS^1^
Mean blood loss volume%	46.8 ±3.4	47.4 ±4.0	NS^1^
Transfusion requirement - no. (%)	7 (78)	5 (63)	NS^2^

^1^ Student *t-*test; ^2^ chi-squared; NS: not significant.

**Figure 2:**
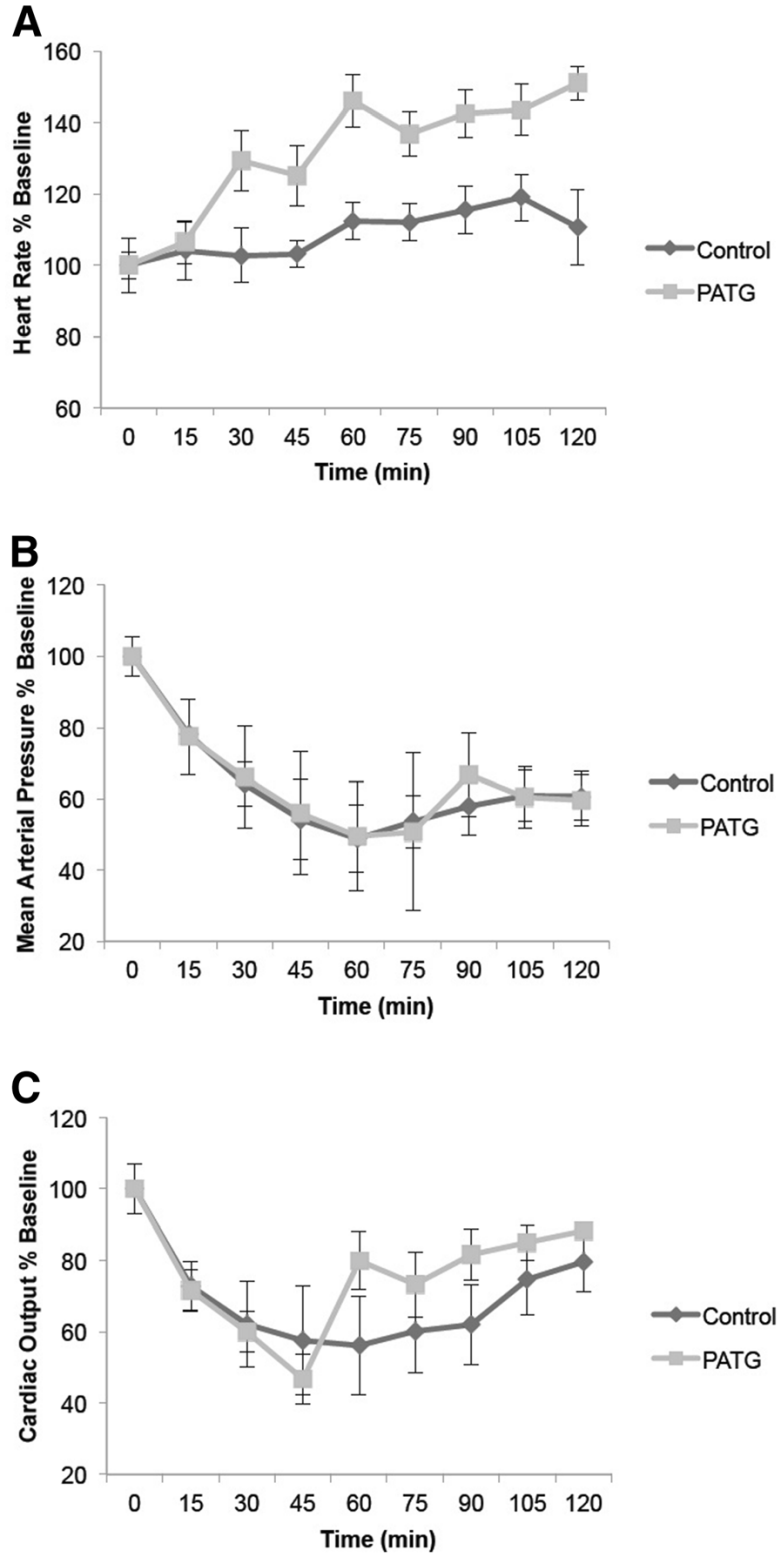
**Hemodynamic profiles during hemorrhage period for experimental and control groups. A)** Heart rate. **B)** Mean arterial pressure. **C)** Cardiac output.

Invasive hemodynamic monitoring during the hemorrhage period demonstrated shock physiology in all animal groups (Figure 2). Cardiac output and mean arterial pressure were significantly decreased and heart rate significantly increased compared to baseline. The heart rate increased markedly in the PATG group, possible due to cytokine release phenomena.

### Lymphocyte depletion significantly improved reperfusion survival

**Figure 3:**
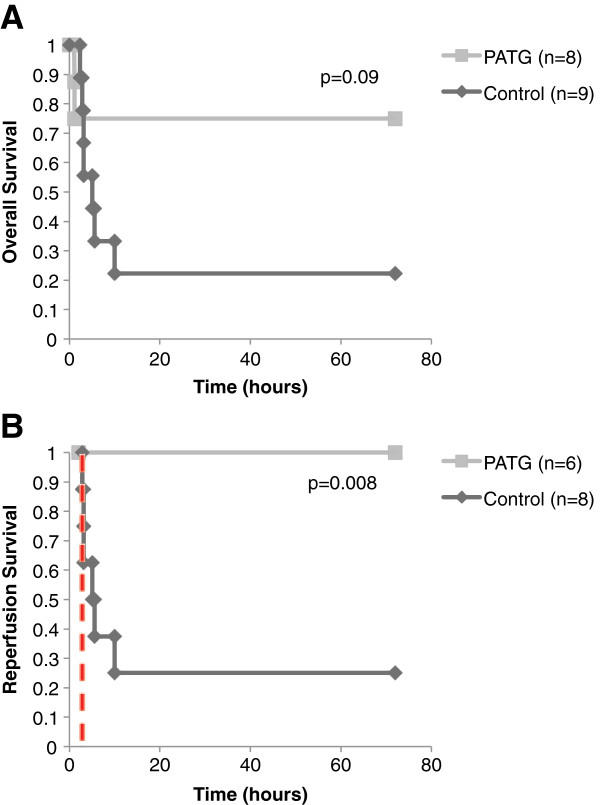
**Kaplan-Meier survival curve for experimental groups versus control with log-rank test for statistical comparison. A)** Overall survival. **B)** Reperfusion period survival. Dashed red line indicates end of hemorrhage and beginning of reperfusion period.

Overall survival in the experimental group was improved compared to control, although not statistically significant by log rank test (Figure 3A). The control group survival was 22% (2/9) and PATG was 75% (6/8) p = 0.09. During the reperfusion period (following hemorrhage) survival was statistically improved in the experimental group compared to control (Figure 3B). Control group survival was 25% (2/8) and PATG was 100% (6/6) p = 0.008 during reperfusion.

### Peripheral and central lymphocyte response to hemorrhagic shock

**Figure 4:**
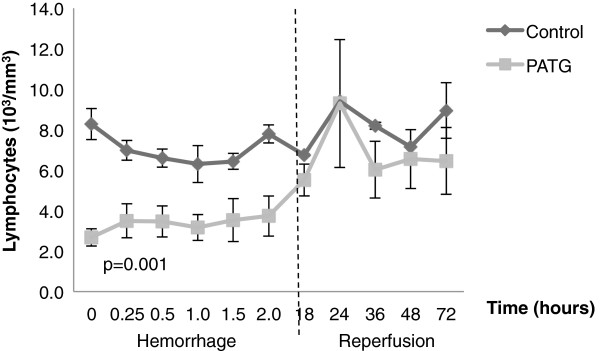
**Peripheral lymphocyte counts during hemorrhage and reperfusion periods.** One-way ANOVA with repeated measures design was used for statistical comparison. Error bars represent ± SEM.

We examined the peripheral, or circulating, and central lymphocyte response to hemorrhagic shock in the experimental and control groups. Peripheral lymphocyte counts were significantly decreased in the PATG group during the hemorrhage period compared to control, p = 0.001, indicating effective lymphocyte depletion (Figure 4). During the reperfusion period, peripheral lymphocyte counts in the PATG group were not significantly different from control.

**Figure 5:**
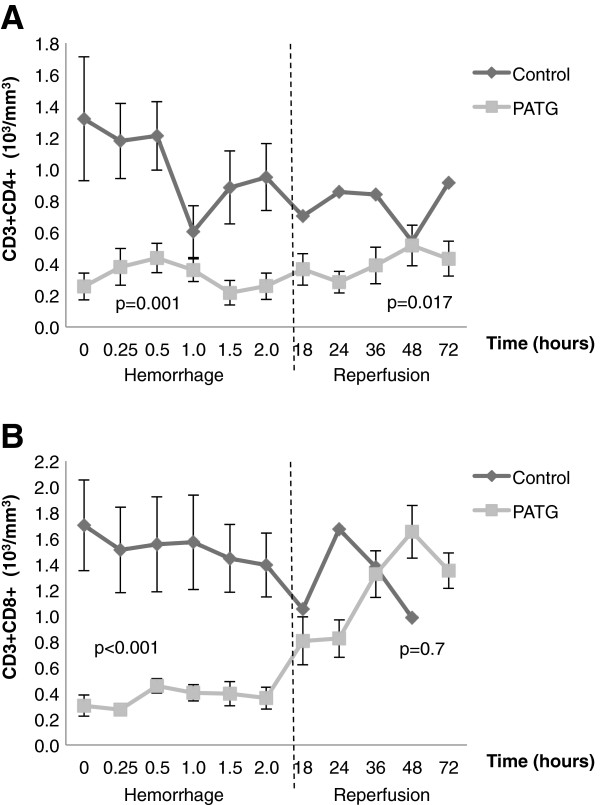
**CD3**^**+**^**CD4**^**+ **^**and CD3**^**+**^**CD8**^**+ **^**lymphocyte counts were quantified throughout hemorrhage and reperfusion A) Quantification of CD3**^**+**^**CD4**^**+ **^**and B) CD3**^**+**^**CD8**^**+ **^**lymphocytes throughout the experiment.** One-way ANOVA with repeated measures design was used for statistical comparison. Error bars represent ± SEM.

CD3^+^CD4^+^ and CD3^+^CD8^+^ lymphocyte counts were quantified throughout hemorrhage and reperfusion periods (Figure 5A, B). During the hemorrhage period, both CD3^+^CD4^+^ (p = 0.001) and CD3^+^CD8^+^ (p < 0.001) lymphocytes were significantly reduced, indicating sustained depletion. During reperfusion, CD3^+^CD4^+^ (p < 0.001) lymphocytes remained significantly depleted, but CD3^+^CD8^+^ (p = 0.7) lymphocytes were not different from controls.

**Figure 6:**
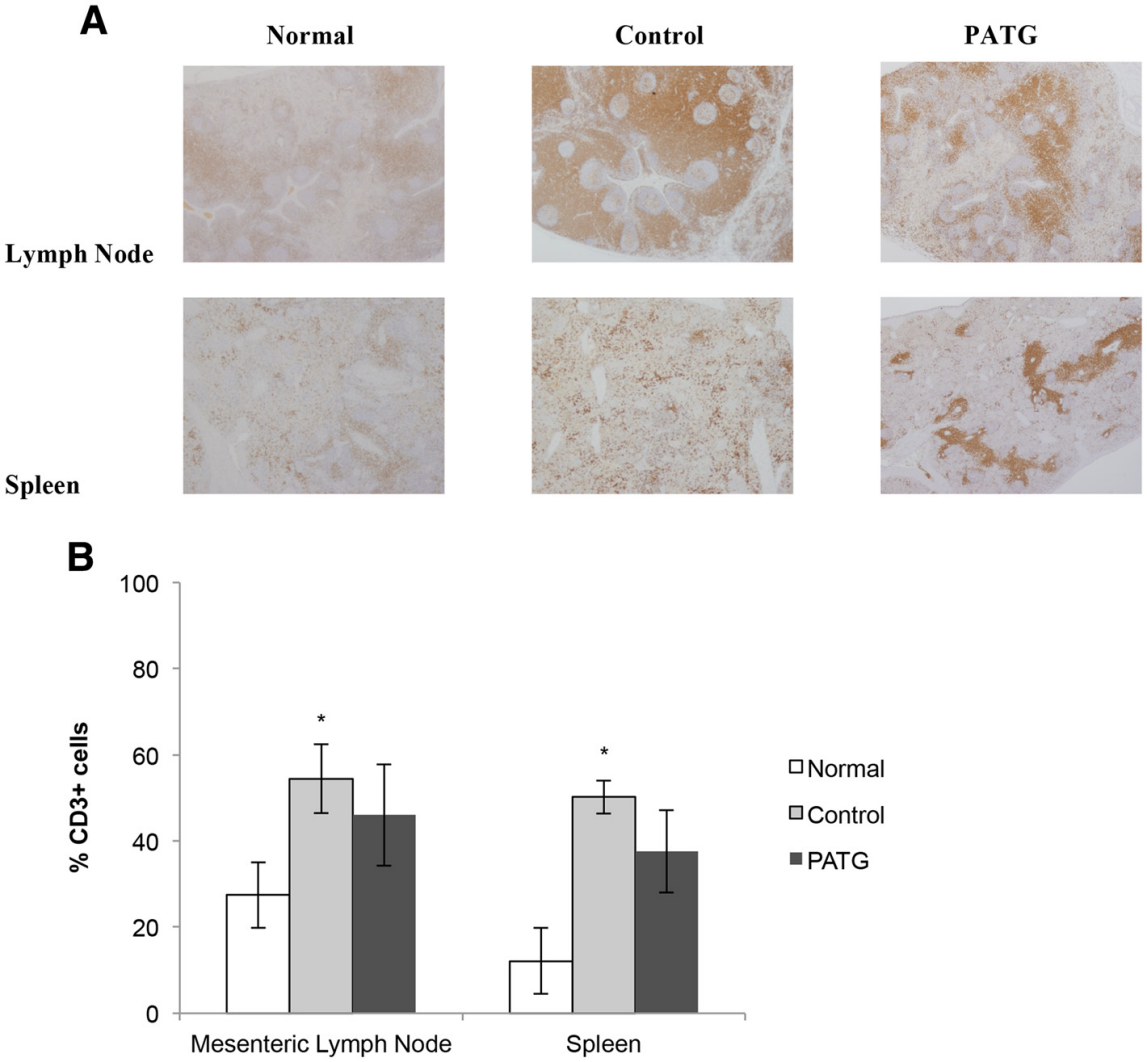
**Central lymphocyte counts at time of necropsy. A)** Representative immunohistochemistry images ofanti- CD3 stains for normal, control, and PATG tissue. **B)** Quantification of CD3^+^ reactivity by automated cellular Imaging. * indicates p < 0.05 compared to normal, unmanipulated tissue. Data is depicted as mean ± SEM.

Central lymphocytes were evaluated with CD3 immunohistochemistry staining of mesenteric lymph nodes and spleen tissue at time of necropsy (Figure 6A). The CD3 staining was quantified, and in the PATG group, central T-cells were decreased compared to control but this did not reach statistical significance. Interestingly, significant central T-cell sequestration was evident in the control group compared to normal, unmanipulated mesenteric lymph node and spleen tissue (p < 0.05). This finding suggests that some degree of lymphocyte sequestration is part of the normal response to hemorrhagic shock.

Altogether, PATG appeared to primarily affect peripheral lymphocytes, and in particular CD4^+^ T-cells, under the experimental conditions.

### Peripheral and tissue neutrophil response to hemorrhagic shock

**Figure 7:**
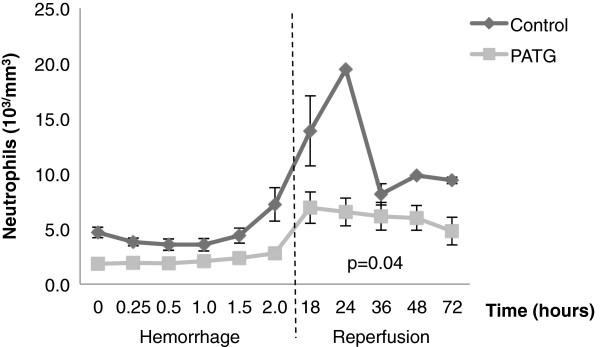
**Peripheral neutrophil counts during hemorrhage and reperfusion periods.** One-way ANOVA with repeated measures design was used for statistical comparison. Error bars represent ± SEM.

In order to study the innate cellular response to hemorrhagic shock, we examined peripheral and tissue neutrophils in the experimental and control groups. Throughout the hemorrhage period, there was some evidence of peripheral neutrophil depletion in the PATG group, although not statistically significant when compared to control (Figure 7). During reperfusion, peripheral neutrophils were markedly increased in the control group, particularly during the peak reperfusion injury period (t = 24 hours). This peripheral neutrophil response was attenuated in the PATG (p = 0.04) group throughout the reperfusion period.

**Figure 8:**
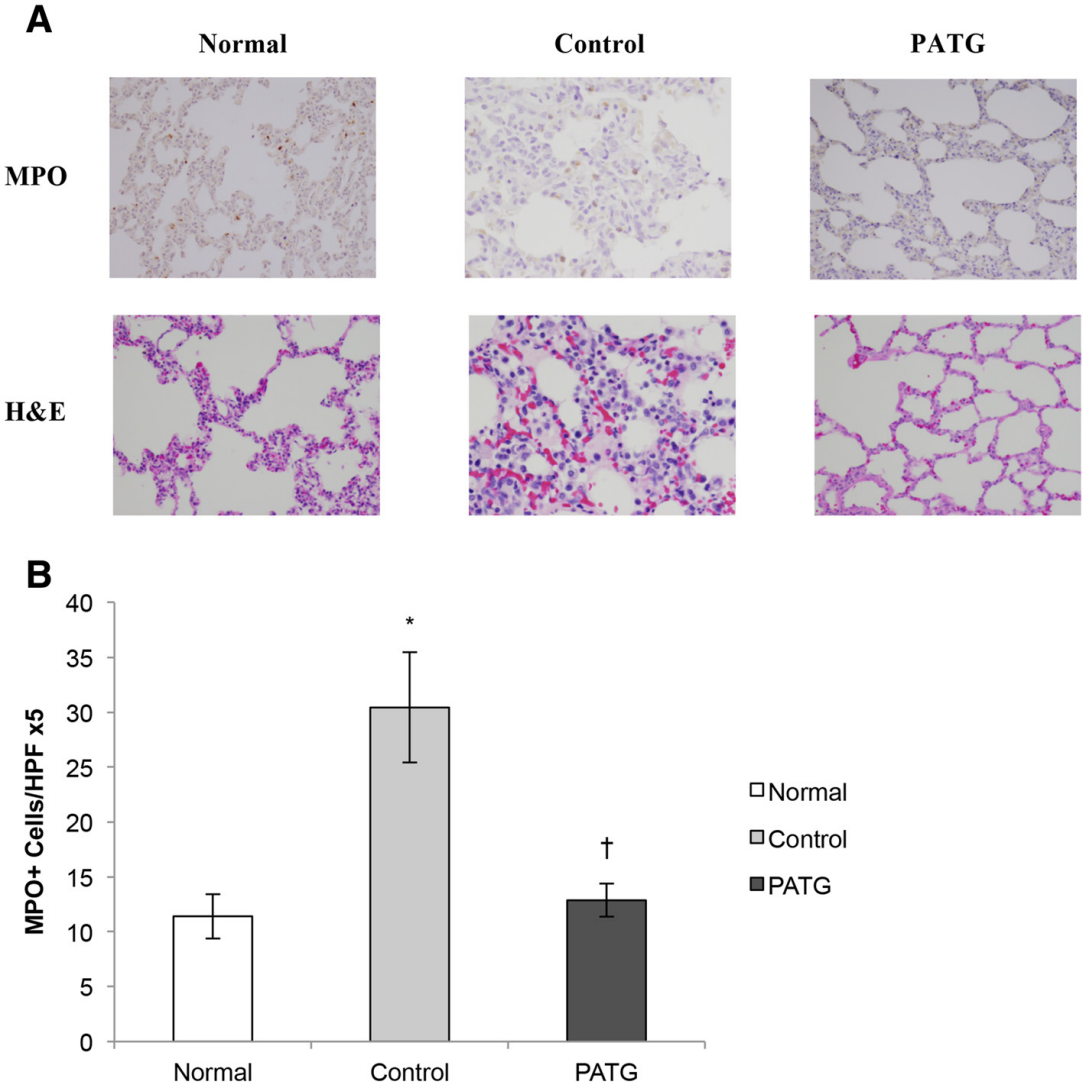
**Lung tissue neutrophil counts at time of necropsy.A)** Representative histology pictures of normal, control and PATG tissue. **B)** Quantification of MPO^+^ cell reactivity. * indicates p < 0.05 compared to normal, unmanipulated tissue. † indicates p < 0.05 compared to control group. Data is depicted as mean ± SEM.

The tissue neutrophil response to hemorrhagic shock was evaluated by examining lung tissue neutrophil infiltration, a major target organ of neutrophils following shock [24,25]. There was histologic evidence of significant neutrophil infiltration in control lung tissue (Figure 8A). This was quantified with myeloperoxidase (MPO) staining and control MPO^+^cells were significantly increased compared to normal, unmanipulated lung (Figure 8B). As with the peripheral neutrophils, lung tissue neutrophils were significantly decreased in the PATG (p = 0.003) group compared to control. Thus, both peripheral and tissue neutrophils were attenuated in the experimental group, suggesting that lymphocyte manipulation also disrupted the innate neutrophil response to hemorrhagic shock.

### Immune molecular profile in hemorrhagic shock

**Figure 9:**
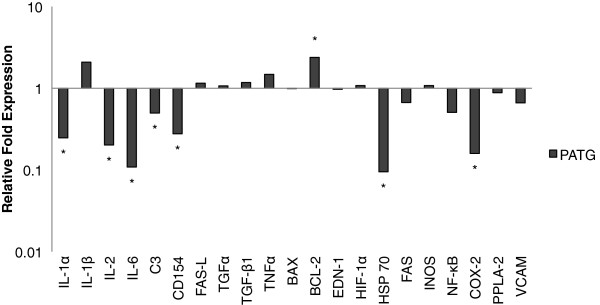
**Gene transcript expression in liver tissue of PATG relative to the control group.** Median value of relative fold expression is depicted on a logarithmic scale. >2 fold difference from control was considered statistically significant and is indicated by *.

We analyzed 21 immune-related gene mRNA transcripts from liver tissue, a major effector organ of the systemic immune response to shock [11]. Multiple immune targets, including IL-1α, IL-2, IL-6, C3, CD154, HSP70 and COX-2, were significantly down-regulated in the PATG group relative to the control group (Figure 9). An apoptosis gene, BCL-2, was up-regulated in the PATG group. Thus, lymphocyte depletion appeared to reduce liver inflammation following hemorrhagic shock.

## Discussion

In this study we demonstrate that lymphocyte depletion significantly improves reperfusion survival following experimental hemorrhagic shock in a clinically relevant swine model. Lymphocyte disruption appears to attenuate innate cellular and molecular activation following hemorrhagic shock, evident by significantly decreased circulating and lung tissue infiltrating neutrophils and decreased expression of liver innate immune-related genes. Lymphocytes mediate critical innate events following hemorrhage, and lymphocyte modulation may ameliorate reperfusion injury.

The innate role of lymphocytes offers the potential for targeting lymphocytes in the setting of severe injury and hemorrhagic shock. While ATG is primarily used clinically to suppress adaptive lymphocyte responses, pre-clinical studies have demonstrated the utility of ATG in abrogating innate immune responses. In a non-human primate model, polyclonal ATG conferred a protective effect on reperfusion injury following limb ischemia [26]. In clinical kidney and liver transplantation, ATG has been shown to reduce graft dysfunction associated with IRI [27]. Specifically, ATG has been shown to reduce delayed graft function following transplantation, an event that is thought to be related to IRI.

Here we corroborate these findings in demonstrating that lymphocyte depletion prior to hemorrhage reduces innate immune-mediated injury during reperfusion. We developed a pig specific ATG by immunizing rabbits with purified populations of porcine thymocytes, similar to Thymoglobulin (Genzyme, Cambridge, MA) currently used in human clinical application [28]. PATG resulted in significant peripheral lymphocyte depletion, which was sustained during experimental hemorrhage. Total lymphocyte counts were not significantly reduced during the reperfusion period compared to control, however CD4^+^ T lymphocytes remained depleted throughout the experiment. Central, including mesenteric lymph node and spleen, T lymphocytes were not significantly depleted in PATG animals compared to control. This may be an effect of hemorrhage on lymphocyte trafficking, reducing the effect of PATG mediated depletion. Depletion of primarily circulating lymphocytes, and in particular CD4^+^ T lymphocytes, appeared to be responsible for reduced immune injury following hemorrhagic shock.

The significant depletion of CD4^+^ T lymphocytes in the PATG group suggests a critical role for the CD4^+^ T lymphocytes in modulating innate immune responses to hemorrhage. The specific characterization of CD4^+^ T lymphocytes in IRI has been demonstrated in several animal studies. In an elegant experiment, Rabb and colleagues demonstrated that knockout mice deficient in CD4^+^ T lymphocytes, but not mice deficient in CD8^+^ T lymphocytes, were significantly protected in a renal IRI model [10]. Adoptive transfer of wild-type CD4^+^ lymphocytes into these knockout mice restored the injury phenotype, directly demonstrating the importance of CD4^+^ lymphocytes in IRI. Thus, the pathogenic role of CD4^+^ T lymphocytes in IRI may account for the immune protection in the PATG group following hemorrhage.

Neutrophils are the earliest cellular mediators of IRI following hemorrhage [29-31]. In particular, neutrophils have been shown to mediate acute lung injury following hemorrhagic shock, contributing to acute respiratory distress syndrome, a significant etiology of morbidity and mortality after trauma [24,25]. Recent mechanistic studies have demonstrated that lymphocytes may coordinate innate neutrophil action following ischemic injury. In an intestinal IRI model, T lymphocytes were shown to orchestrate neutrophil trafficking [11]. In the current study, lymphocyte depletion resulted in a significant decrease in circulating and tissue infiltration of neutrophils. The reduction in lung tissue neutrophil infiltration likely contributed to the improved survival in the PATG treated animals. Innate lymphocyte and neutrophil function may be intimately linked, and lymphocyte disruption in the setting of hemorrhage appears to abrogate neutrophil mediated immune injury.

Following hemorrhagic shock and severe injury, the liver is responsible for initiating and sustaining the acute inflammatory response [7]. Inflammatory mediators released by the liver, including cytokines and chemokines, direct the innate cellular response to IRI. We studied this liver inflammatory response following hemorrhagic shock with relevant immune gene expression. Inflammatory mediator expression, including IL-1a, IL-2, IL-6, C3, CD154, HSP70, and COX-2, was significantly reduced in the PATG treated animals. While Kupffer cells and neutrophils are thought to be the cells responsible for initiating and propagating the injury response, there is an emerging view that both circulating and resident T-cells also regulate liver IRI. Circulating CD4^+^ T lymphocytes have been shown to rapidly recruit to the liver within 1 hour following IRI, and contribute to the early inflammatory mediator release [7]. In addition, in a mouse liver ischemia model, a marked preservation of liver function was found in T lymphocytes deficient athymic (*nu/nu*) mice compared to wild type mice [12]. In our study, depletion of T lymphocytes with PATG appeared to attenuate the liver specific inflammatory response, further validating the lymphocyte as a therapeutic target for immunomodulation following shock.

An important limitation in the interpretation of this study is the multitude of antibody specificities in polyclonal ATG preparations [32]. In addition to lymphocyte depletion, ATG also interferes with leukocyte-endothelium interactions by binding to adhesion molecules and chemokine receptors [33], and these effects may account for some of the observed immune protection in the experimental group. Furthermore, ATG has B lymphocyte depletional effects [34], while we focused primarily on the contribution of only T-cell. B-cells have been shown to prevent IRI in multiple models and may play an important role in the post-injury immune response [35,36]. In addition, a relatively high dose of PATG was required for >50 percent lymphocyte depletion. High ATG doses can result in hemolytic anemia, neutropenia, and thrombocytopenia [18]. A non-statistically significant reduction in neutrophils was observed in the experimental group, which may nonetheless confound the study results.

The current experiment is a proof of principle study, as PATG was administered to animals prior to hemorrhage, rather than after injury. We are currently investigating the role of PATG administration following hemorrhage, however preliminary results suggest that the aforementioned cytokine release phenomena results in prohibitive hemodynamic changes in the setting of ongoing hemorrhage. Regardless, there is a large array of pharmacologic strategies currently available that target lymphocyte function, primarily for clinical use in the setting of transplantation and autoimmune diseases. Humanized monoclonal antibodies, such as anti-CD52 monoclonal antibody (alemtuzumab), result in less cytokine release, and may be clinically applicable following significant hemorrhage or injury [37]. Importantly, the identification of lymphocytes as important components of the innate immune system may lead to harnessing advancements in lymphocyte biology for novel hemorrhagic shock therapies. We are currently exploring these potential therapeutic pathways and have recently demonstrated the efficacy of FTY720, a lymphocyte sequestration agent, in our hemorrhagic shock model [38].

## Conclusion

In conclusion, we demonstrate that lymphocyte depletion significantly improves reperfusion survival in a large animal hemorrhagic shock model. PATG resulted in depletion of primarily circulating T lymphocytes, and T lymphocyte disruption appeared to attenuate innate cellular and molecular activation following hemorrhagic shock. This proof of principle study paves the way for further development of immunomodulation approaches to ameliorate reperfusion injury in trauma and surgical patients.

## Competing interests

The authors declare that they have no competing interests.

## Authors’ contributions

Conceived and designed the experiments: EE DT. Performed the experiments: JSH JCG SG TB. Analyzed the data: JSH JCG TB SW TD EE JSH SG. Wrote the paper: JSH JCG. Supervised the protocol that obtained the information that lead to the writing of the manuscript: TB TD DT EE. Final revisions of the manuscript: DT TB TD EE. All authors read and approved the final manuscript
